# GBP2 promotes M1 macrophage polarization by activating the notch1 signaling pathway in diabetic nephropathy

**DOI:** 10.3389/fimmu.2023.1127612

**Published:** 2023-08-09

**Authors:** Xiaohui Li, Jialu Liu, Mengru Zeng, Kexin Yang, Shumin Zhang, Yifei Liu, Xiangxiang Yin, Chanyue Zhao, Wenpeng Wang, Li Xiao

**Affiliations:** Department of Nephrology, Hunan Key Laboratory of Kidney Disease and Blood Purification, The Second Xiangya Hospital, Central South University, Changsha, China

**Keywords:** diabetic nephropathy, bioinformatics analysis, GBP2, M1 macrophage, inflammation

## Abstract

**Background:**

Diabetic nephropathy (DN) is one of the most common diabetic complications, which has become the primary cause of end-stage renal disease (ESRD) globally. Macrophage infiltration has been proven vital in the occurrence and development of DN. This study was designed to investigate the hub genes involved in macrophage-mediated inflammation of DN via bioinformatics analysis and experimental validation.

**Methods:**

Gene microarray datasets were obtained from the Gene Expression Omnibus (GEO) public website. Integrating the CIBERSORT, weighted gene co-expression network analysis (WGCNA) and DEGs, we screened macrophage M1-associated key genes with the highest intramodular connectivity. Subsequently, the Least Absolute Shrinkage and Selection Operator (LASSO) regression was utilized to further mine hub genes. GSE104954 acted as an external validation to predict the expression levels and diagnostic performance of these hub genes. The Nephroseq online platform was employed to evaluate the clinical implications of these hub genes. Gene Ontology (GO) and Kyoto Encyclopedia of Genes and Genomes (KEGG) were performed to elucidate the dominant biological functions and signal pathways. Finally, we conducted experiments to verify the role of GBP2 in M1 macrophage-mediated inflammatory response and the underlying mechanism of this role.

**Results:**

Sixteen DEGs with the highest connectivity in M1 macrophages-associated module (paleturquoise module) were determined. Subsequently, we identified four hub genes through LASSO regression analysis, including CASP1, MS4A4A, CD53, and GBP2. Consistent with the training set, expression levels of these four hub genes manifested memorably elevated and the ROC curves indicated a good diagnostic accuracy with an area under the curve of greater than 0.8. Clinically, enhanced expression of these four hub genes predicted worse outcomes of DN patients. Given the known correlation between the first three hub genes and macrophage-mediated inflammation, experiments were performed to demonstrate the effect of GBP2, which proved that GBP2 contributed to M1 polarization of macrophages by activating the notch1 signaling pathway.

**Conclusion:**

Our findings detected four hub genes, namely CASP1, MS4A4A, CD53, and GBP2, may involve in the progression of DN via pro-inflammatory M1 macrophage phenotype. GBP2 could be a promising prognostic biomarker and intervention target for DN by regulating M1 polarization.

## Introduction

Diabetic nephropathy (DN), known as one of the most prevalent diabetic microvascular complications, has become the main cause of end-stage renal disease (ESRD) (1). However, current treatments including blood glucose, blood pressure, and albuminuria control remain suboptimal in delaying DN evolution into ESRD. Therefore, continued shedding light on the pathogenesis mechanisms of DN is of great significance to develop new effective treatments.

Mounting evidence demonstrates that inflammatory disorders, especially tubulointerstitial macrophage infiltration, play an indispensable role in the onset and progression of DN. Macrophages have been detected in renal tissues of experimental animal models as well as patients at the early stage of diabetic nephropathy (2, 3). Furthermore, the level of tubulointerstitial macrophage accumulation parallels renal dysfunction and interstitial fibrosis (4, 5). In turn, the specific removal of macrophages by clodronate liposomes or treating transgenic CD11b- diphtheria toxin (DT) receptors (DTR) mice with DT mitigated pathological damage of DN (6, 7). According to the activation model and biological functions, macrophages can differentiate into pro-inflammatory M1 phenotype and anti-inflammatory M2 phenotype. The former is classically activated and secretes inflammatory factors, and the latter is alternatively activated and produces an anti-inflammatory effect. After injury onset, M1 macrophage is generally present at the early stage of inflammation and contributes to tissue injury, whereas M2 macrophage participates in the repair phase and provides cytokines that suppress inflammation (8). Thus, the well-maintained balance between M1 macrophage and M2 macrophage is essential to tissue repair. If the M1 phenotype persists or the M2 phenotype decreases, fibrosis will occur (9). M1 macrophage is the major macrophage phenotype present in DN and greatly contributes to the progression of DN (7, 10). Therefore, our study aims to explore the hub genes which involve in M1 macrophage polarization for providing novel therapeutic targets.

## Materials and methods

### Data acquisition

The microarray dataset GSE30122 was downloaded from the public online GEO (http://www.ncbi.nlm.nih.gov/geo) database. The data set is based on GPL571 (Affymetrix Human Genome U133A 2.0 Array) containing 10 renal tubulointerstitial tissue samples from DN patients and 24 normal samples. External validation dataset GSE104954 consists of two platforms. The former is GPL22945 which includes 7 DN patients and 18 normal controls. The latter is GPL24120 which contains 10 DN patients and 3 normal controls.

### Data preprocessing and sscreening of DEGs

The batch effect was removed using the SVA package in the R language. Probe annotation was then performed. We removed the probes without corresponding gene symbols and averaged the probes with multiple gene symbols. The limma R package was employed to determine DEGs by the criteria of |logFC (fold change) | > 1 and adjusted p< 0.05.

### Immune cell infiltration evaluation

With LM22 signature genes as the reference, the CIBERSORT algorithm was applied to calculate the relative proportions of 22 immune cell subtypes in each sample.

### Weighted gene correlation network analysis construction and trait-related module identification

Based on the detected genes, the WGCNA package of R software was utilized to conduct a weighted co-expression network. The soft-thresholding power β = 18 was determined to establish a scale-free network. To classify genes with similar expression profiles into gene modules, average linkage hierarchical clustering was conducted with a minimum size of 30 genes in each module. As a result, 16 modules were obtained. Subsequently, the correlation between each module and infiltrating immune cells was performed using Pearson’s correlation analysis. Among these 16 modules, the paleturquoise module possesses the highest correlation with M1 macrophages and was used for hub genes identification.

### Functional enrichment analysis

To demonstrate the dominant biological function and signal pathways of the identified genes, GO terms and the KEGG pathways analysis were conducted using the clusterProfiler package.

### Screening and validation of hub genes

Based on the cut-off criteria (|MM|>0.8 and |GS|> 0.20), genes with the highest connectivity were identified as candidate genes. The intersection of candidate genes and DEGs was regarded as hub genes and was subjected to GO and KEGG analysis. Subsequently, these identified hub genes were exposed to the Least absolute shrinkage and selection operator (LASSO) analysis for final hub genes screening. The levels of hub genes were displayed by the ggplot2 package. QROC package in R was applied to estimate the diagnostic performance of these hub genes according to the area under the curve (AUC). GSE104954 was used as an external dataset to further verify the expression of hub genes and their diagnostic accuracy.

### The clinical significance of these identified hub genes

We assessed the correlation between hub genes and clinical indicators which reflected the severity and prognosis of DN through the Nephroseq online tool.

### Immunohistochemistry staining

Paraffin sections were first deparaffinized and rehydrated and subjected to antigen retrieval with citrate buffer. After blocking, sections were incubated overnight at 4°C in the anti-GBP2 primary antibody (11854-1-AP, Proteintech, 1:200) or anti-F4/80 primary antibody (GB113373, Servicebio, 1:200) followed by incubation with secondary antibody conjugated with HRP for 1 h at room temperature. The antigen-antibody reaction was visualized with Diaminobenzidine (DAB, Invitrogen). The nuclei were marked with hematoxylin. Images were captured using a Nikon microscope and processed using Image Pro Plus software (version 6.0).

### Immunofluorescence staining

Frozen kidney tissues were sliced at a 5um thickness and fixed in methanol at -20 °C for 10 minutes. After being washed 3 times with phosphate-buffered saline (PBS), tissue sections were blocked in 5% BSA for 30 minutes. Subsequently, they were subject to rabbit anti-GBP2 (11854-1-AP, Proteintech, 1:100) and rat anti-F4/80 (ab6640, Abcam, 1:200) overnight at 4°C, which was followed by incubation with fluorescein isothiocyanate (FITC)-labeled secondary antibody in the dark for 1 h at 37°C and 4,6-Diamidino-2-phenylindole (DAPI, SouthernBiotech) for nuclear counterstaining. Slides were observed and images were captured using a fluorescence microscope (DMI-3000B, Leica, Germany).

### Cell culture and treatment

THP-1 cells (ATCC) were maintained in Roswell Park Memorial Institute (RPMI) 1640 medium (Gibco, USA)) supplemented with 10% fetal bovine serum (FBS) (Invitrogen) in a 5% CO2 atmosphere at 37°C. THP-1 cells were stimulated with 100 ng/mL PMA (Sigma) for 24 h to induce resting (M0) macrophages, which was followed by incubating with 30mM D-glucose (Sigma) or 100 ng/ml LPS (Sigma) for 24 h. GBP2 siRNA was transfected into THP-1 cells with Lipofectamine 2000 reagent according to the manufacturer’s instructions.

### Western blot assay

The total protein was extracted from THP-1 cells with RIPA lysis buffer plus 1% protease inhibitor and 1% phosphatase inhibitor. A BCA protein assay kit was applied to quantify protein concentrations. Protein samples were then mixed with 5×loading buffer and separated through SDS-PAGE. Subsequently, proteins were transferred onto a PVDF membrane, blocked in 5% skim milk and blotted with different primary antibodies overnight at 4 °C. These primary antibodies included anti-GBP2 (11854-1-AP, Proteintech, 1:1000), anti-NOS (D6B6S, Cell Signaling Technology, 1:1000), TNF-α (ab6671, Abcam, 1:1000), Notch1 (ab52627, Abcam, 1:1000), N1ICD (ab52301, Abcam, 1:1000), and GAPDH (10494-1-AP, Proteintech, 1:1000). Horseradish peroxidase-conjugated anti-mouse and anti-rabbit antibodies served as secondary antibodies. Western blots were developed using ECL reagent and the expression of protein was quantified using Image J.

### RNA extraction and quantitative RT-PCR

Total RNA was extracted from THP-1 cells with TRIzol (Takara) and reverse transcribed to cDNA by a PrimeScriptRT reagent kit (Takara). To estimate the relative transcript levels of genes, real-time quantitative PCR was carried out using a Light Cycler 96 System (Roche). We used SYBR Green for the fluorescent dye. The cycling conditions were set according to the instruction. Each experiment was carried out in three independent reactions. Relative mRNA expression was normalized by GAPDH and the result was analyzed using the 2-ΔΔCt method. One-way analysis of variance was used for the statistical analysis, and P <0:05 indicated a significant difference. The sequences of RNA primers were provided in **Table 1**.

**Table 1:** Primers involved in RT-qPCR.

Gene	Species	Primer	Sequence
CCL2	Human	forward	5′-AGAATCACCAGCAGCAAGTG-3′
		reverse	5′-TCCATGGAATCCTGAACCCA-3′
CD206	Human	forward	5′-GCGGAACCACTACTGACTA-3′
		reverse	5′-GTTGTTGGCAGCTTTTCCTC-3′
Arg1	Human	forward	5′- ACTAGGAAGAAAGAAAAGG-3′
		reverse	5′- TCTTCTGTGATGTAGAGACC-3′
IL-10	Human	forward	5′- AGAACCTGAAGACCCTCAGGC-3′
		reverse	5′- CCACGGCCTTGCTCTTGTT-3′
CD163	Human	forward	5′- CCAACAAGATGCTGGAGTGAC-3′
		reverse	5′- TGACAGCACTTCCACATTCAAG-3′
GAPDH	Human	forward	5′-ATGACATCAAGAAGGTGGTG-3′
		reverse	5′-CATACCAGGAAATGAGCTTG-3′

## Results

### Identification of DEGs and enrichment analysis

**Figure 1:**
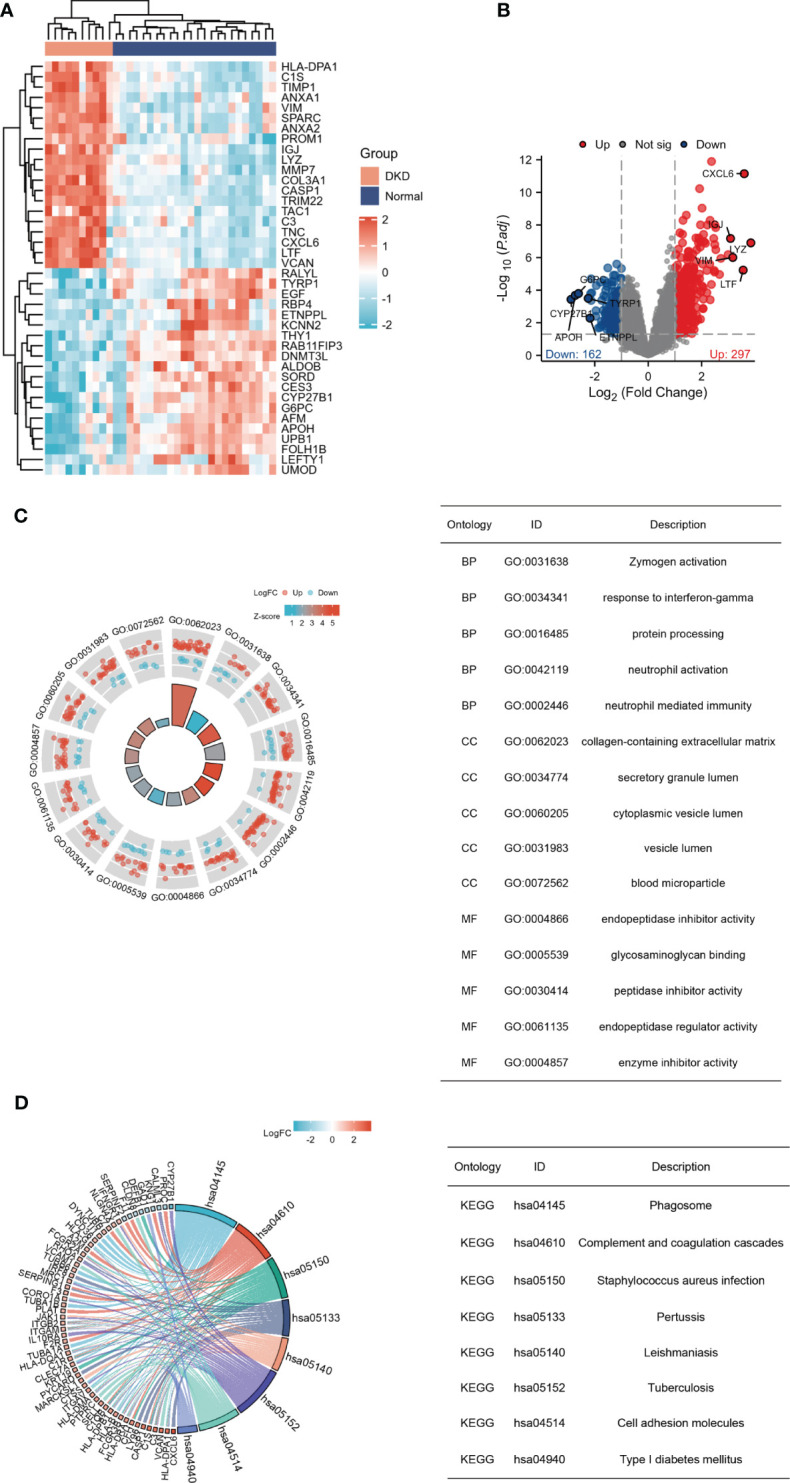
Recognition and enrichment analysis of DEGs expressed in the tubulointerstitium between diabetic nephropathy patients and normal individuals. **(A)** Heatmap for the top 20 DEGs between DN and normal samples. **(B)** Volcano plot of DEGs between DN and normal controls. Red denotes up-regulation and blue denotes down-regulation. **(C)** GO functional annotation demonstrates the top 15 GO terms. **(D)** KEGG pathway enrichment analysis demonstrates the top 8 pathways. DEGs, differentially expressed genes; GO, gene ontology; KEGG, Kyoto Encyclopedia of Genes and Genomes.

A total of 459 genes, consisting of 297 up-regulated genes and 162 down-regulated genes, were screened (**Figures 1A, B**). GO enrichment analysis was conducted to determine the main biological functions these DEGs participated in. Enriched cell components were associated with collagen-containing extracellular matrix and secretory granule lumen. Enriched biological processes were associated with zymogen activation, response to interferon-gamma, and protein processing. Enriched molecular functions were related to endopeptidase inhibitor activity and glycosaminoglycan binding (**Figure 1C**). KEGG pathway analysis revealed that phagosome, complement and coagulation cascades, and staphylococcus aureus infection were prominent enrichment pathways (**Figure 1D**). The enrichment analysis revealed a close connection between DEGs and immune response.

### Immune cell infiltration

**Figure 2:**
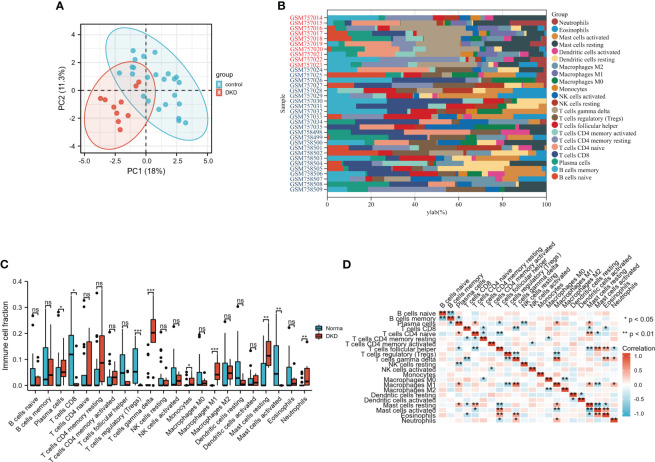
Analysis of immune cell infiltrates using CIBERSORT algorithm. **(A)** The distribution of infiltrating immune cells between two groups by principal component analysis (PCA). **(B)** The composition of 22immune cell subtypes in the DN and control samples. Red denotes DN samples and blue denotes control samples. **(C)** Contrast of immune infiltrating cells between DN and control group through Wilcox rank-sum test. **(D)** Correlation heatmap of 22 kinds of immune cells. Red denotes positive correlations, while blue denotes negative correlations. *P < 0.05; **P <0.01; ***P <0.001. The “ns” indicates “no statistical difference”.

To observe infiltrating immune cells in DN and normal human kidneys, CIBERSORT was applied to evaluate the distribution of 22 cell types. Firstly, principal component analysis (PCA) was conducted to elucidate the difference in infiltrating immune cells between DN and normal samples. The result indicated that the relative proportions of immune cells in tissues from DN patients manifested distinguishable from normal controls (**Figure 2A**). The histograms demonstrated the enrichment fraction of 22 types of immune cells in the control and DN samples (**Figure 2B**). Immune infiltration analysis elucidated increases in the numbers of M1 macrophages, monocytes, neutrophils, resting mast cells, plasma cells, and γδ T cells, whereas activated mast cells, CD8+ T cells, and regulatory T cells were significantly down-regulated in the renal tubulointerstitium of DN patients (**Figure 2C**). Furthermore, M1 macrophages were strongly and positively associated with γδ T cells. Activated mast cells negatively correlated with resting mast cells. A negative correlation between memory B cells and naïve B cells was also obtained (**Figure 2D**). Immune infiltration analysis confirms that inflammatory cells, including M1 macrophages, exist in DN and chronic inflammation plays a vital role in the pathogenesis of DN (11, 12).

### Construction of the weighted co-expression network and identification of M1 macrophages-related module

**Figure 3:**
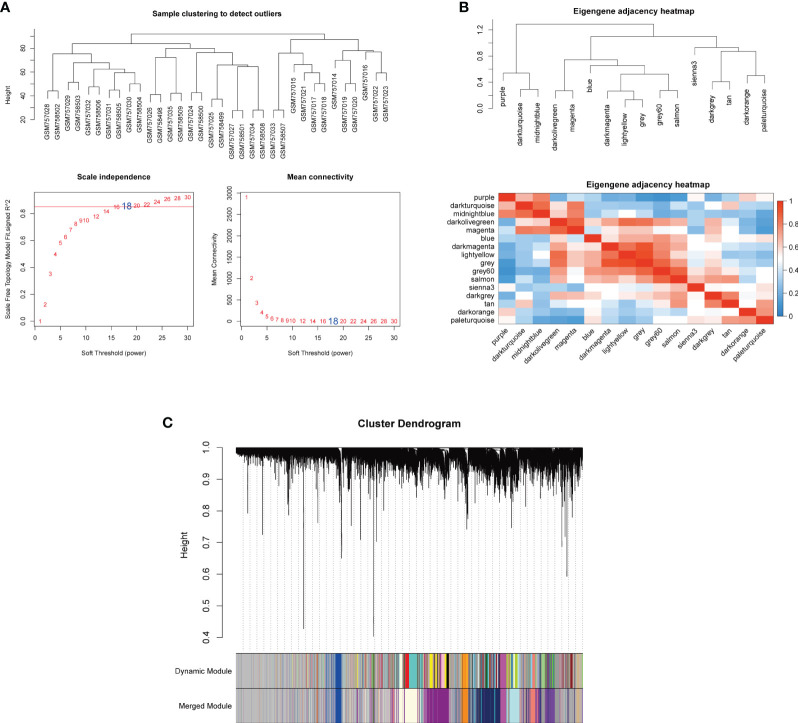
Co-expression network based on WGCNA. **(A)** Sample clustering and selection of optimal soft threshold power β. A soft threshold of 18 was chosen to simultaneously achieve the approximate scale-free topology fit index (R^2^ > 0.80) and optimal mean connectivity. **(B)** Clustered heat maps between modules. **(C)** The cluster dendrogram to identify co-expression modules. Each branch indicates a gene, and the different colors below indicate different co-expression modules. WGCNA, weighted gene co-expression network analysis.

**Figure 4:**
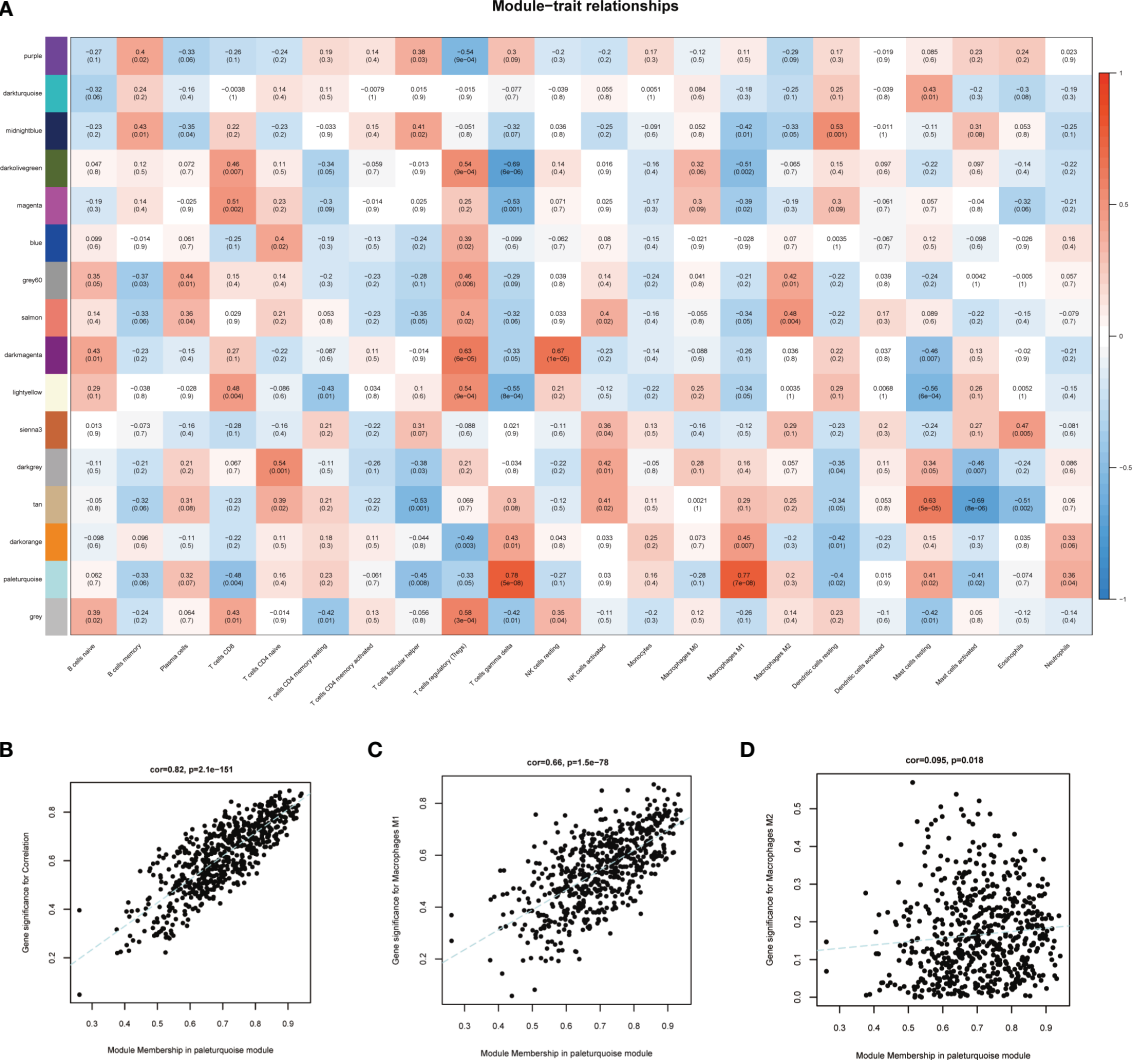
Identification of key module. **(A)** Module-trait correlations between co-expression modules and immune cells. **(B)** Scatter plot displayed the correlation of module membership in the paleturquoise module with gene significance. **(C)** Scatter plot for correlation between paleturquoise module and M1 macrophages (correlation index = 0.66, P =1.5E–78). **(D)** Scatter plot for correlation between paleturquoise module and M2 macrophages (correlation index = 0.095, P=0.018).

To select MI macrophage-associated genes, the WGCNA package of R software was used to construct a weighted co-expression network for all the detected genes. The soft-thresholding power β = 18 was determined to establish a scale-free network (scale-free R^2^ > 0.80), which was based on scale independence and mean connectivity (**Figure 3A**). To classify genes with similar expression profiles into gene modules, average linkage hierarchical clustering was conducted with a minimum size of 30 genes in each module. As a result, 16 modules were obtained (**Figures 3B, C**). Different modules were displayed in different colors, and genes in the same module usually have similar functions. Subsequently, the correlation between these modules and infiltrating immune cells was performed using Pearson’s correlation analysis. Among these 16 modules, the paleturquoise module possessed the highest correlation with M1 macrophages (*r* = 0.77, *p* = 7e-08) (**Figure 4A**), showing that genes in the module ‘paleturquoise’ were most likely involved in macrophage accumulation in DN. Furthermore, to find the key drivers in turquoise modular, intramodular connectivity was used. According to **Figure 4B**, it can be seen that there was a strong linear trend in the correlation between genes and phenotypes and the correlation between genes and modules, indicating that genes highly related to phenotypes are usually important genes within the corresponding modules of that phenotype. Furthermore, genes strongly associated with M1 macrophages were also crucial genes in modules corresponding to this phenotype (**Figure 4C**), which was not observed in the M2 phenotype (**Figure 4D**). Specifically, the paleturquoise module consisted of 618 genes of which 17 genes with the highest intramodular connectivity were determined as candidate genes through the WGCNA function chooseTopHubInEachModule (**Supplementary Tables S1, S2**).

### M1 macrophages-associated hub genes screening

**Figure 5:**
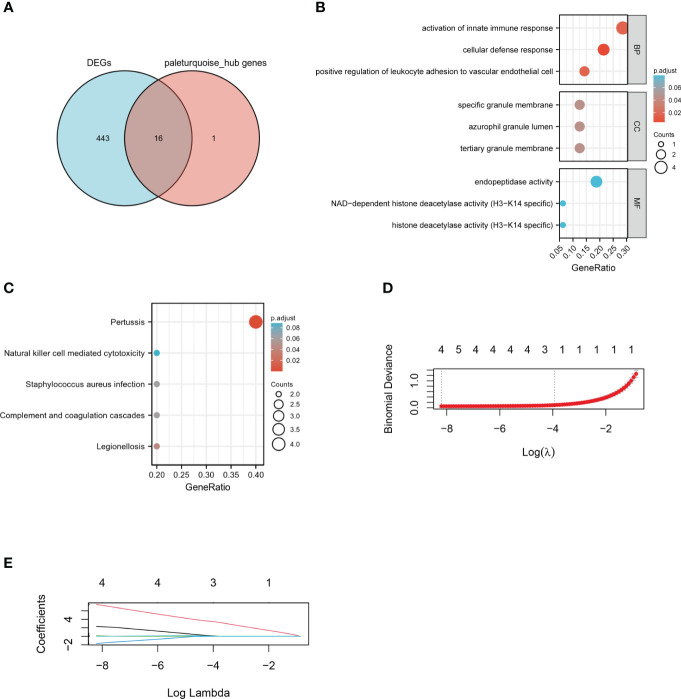
Identification of hub genes. **(A)** Venn diagram of DEGs and key genes in the paleturquoise module. **(B)** GO term analysis of selected hub genes. **(C)** KEGG pathway analysis of selected hub genes. **(D)** The tuning parameter lambda in the Lasso model fits was determined through cross-validation using the glmnet R package. **(E)** The distribution of LASSO coefficient profiles of four hub genes in cross-validation runs. LASSO, least absolute shrinkage and selection operator.

Venn diagram demonstrated 16 intersecting genes based on the overlap between DEGs and hub genes within paleturquoise module (**Figure 5A**). According to go enrichment analysis, these genes were mainly related to cellular defense response, positive regulation of leukocyte adhesion to vascular endothelial cells, activation of innate immune response, and positive regulation of innate immune response (**Figure 5B**). KEGG analysis elucidated the dominant signal pathways were pertussis and legionellosis (**Figure 5C**). The enrichment analysis revealed that these 16 genes were most related to immune processes. Subsequently, the LASSO regression was employed to select the diagnostic features associated with DN. Combining partial likelihood deviance (**Figure 5D**) and coefficient profiles (**Figure 5E**)., 4 diagnostic-related genes, namely CASP1, MS4A4A, CD53, and GBP2, were identified.

### Validation of hub genes expression and identification of diagnostic and prognostic value

**Figure 6:**
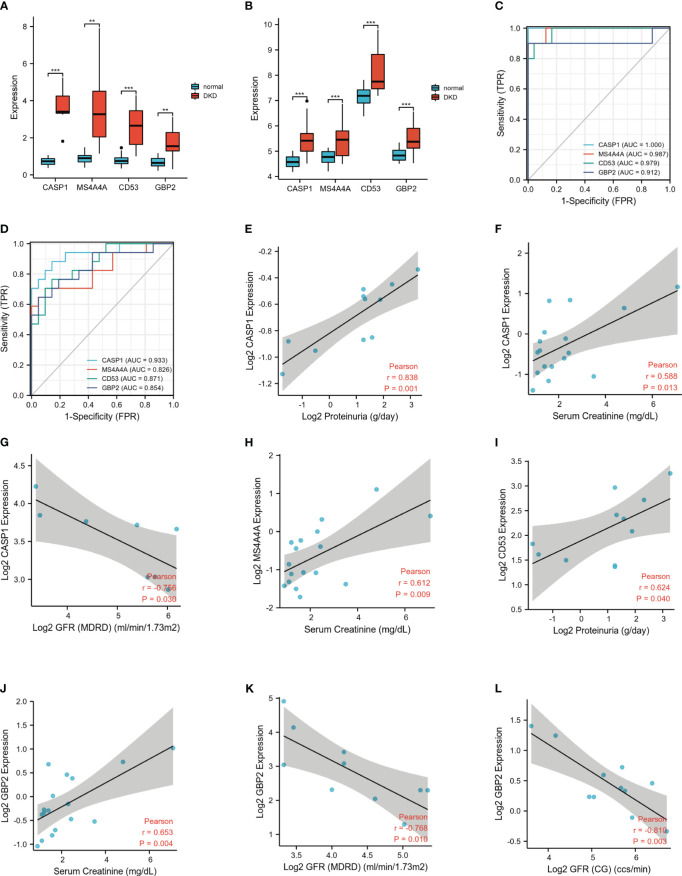
Expression levels and clinical significance of four hub genes. **(A)** Expression of hub genes in GSE30122. **(B)** Similar results were validated in GSE104954. **(C)** The ROC analysis of hub genes in GSE30122. **(D)** The ROC analysis of hub genes in validation dataset GSE104954. **(E-L)**. Correlation between hub genes and clinical characteristics. ROC, receiver operating characteristic. **P <0.01; ***P <0.001.

To further verify the screened 4 genes, GSE104954 was utilized as an external dataset to validate the expression of hub genes and their diagnostic accuracy. Consistently, the levels of all four hub genes expression were obviously increased in renal tubulointerstitial tissues from DN patients compared to control samples (**Figures 6A, B**). According to the ROC curves, all identified hub genes presented with AUC values > 0.90, suggesting a prime diagnostic efficiency for DN (**Figure 6C**). The value of the above-mentioned four hub genes for DN diagnosis was also confirmed, achieving the AUC values > 0.80 in the validation set GSE104954 (**Figure 6D**).

To investigate the prognostic value of these identified hub genes, Nephroseq v5 online tool was used. The increased mRNA expression of MS4A4A in renal tubulointerstium was positively correlated with serum creatine. Up-regulated CD53 mRNA positively correlated with proteinuria. The highly expressed CASP1 positively correlated with serum creatine and proteinuria, inversely correlated with GFR in DN patients. Similarly, the elevated mRNA expression of GBP2 positively correlated with serum creatine and reversely correlated with GFR in DN patients (**Figures 6E-L**). The above evidence demonstrated that high expression of these hub genes may promote the progression of DN and indicate a poor prognosis of DN patients.

### GBP2 drives M1 polarization of macrophages by the activation of the notch1 signaling pathway

**Figure 7:**
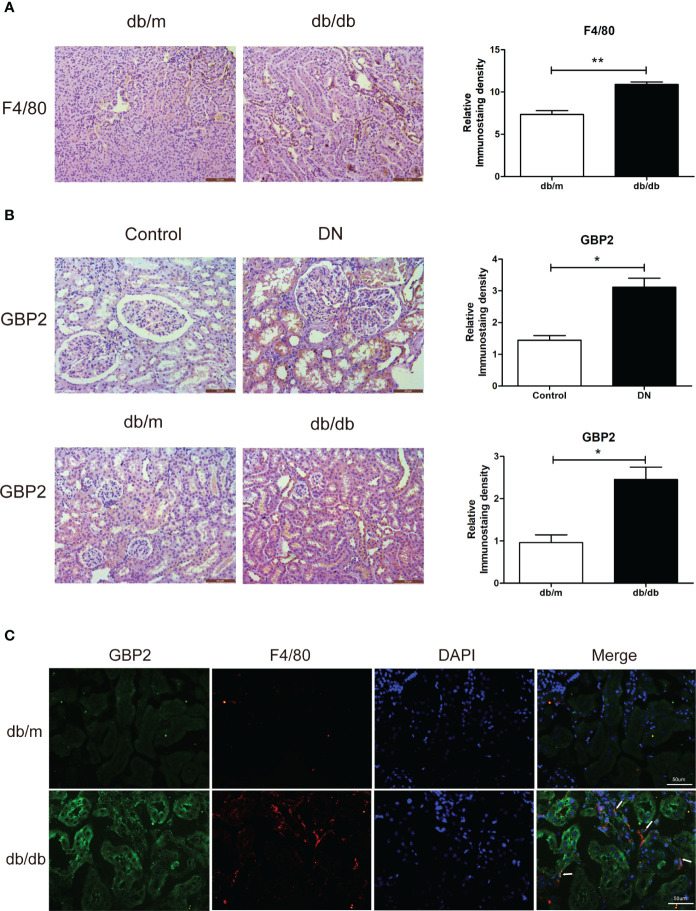
Verification of GBP2 *in vivo*. **(A)** Immunohistochemistry staining of F4/80 in kidney sections from db/m and db/db mice. **(B)** Immunohistochemistry staining of GBP2 in DN and control kidney tissues as well as kidney sections from db/m and db/db mice. **(C)** Immunofluorescence staining of GBP2 (green), macrophage marker F4/80 (red), and DAPI (blue) in frozen kidney sections from db/m mice and db/db mice. Scale bar, 50 um. Data are representative of three independent experiments. Values are expressed as means ± SEM, *P < 0.05; **P <0.01.

**Figure 8:**
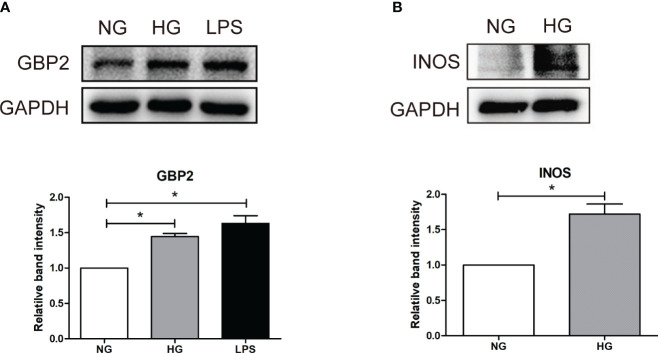
Verification of GBP2 *in vitro*. **(A)** Western blot analysis of GBP2 in THP-1 cells treated with HG (30 mM D-glucose) and LPS (100 ng/ml), and GAPDH was used as an internal control. **(B)** Western blot analysis of M1macrophage markers INOS in THP-1 cells treated with HG (30 mM D-glucose), and GAPDH was used as an internal control. Data are representative of three independent experiments. Values are expressed as means ± SEM, *P < 0.05.

**Figure 9:**
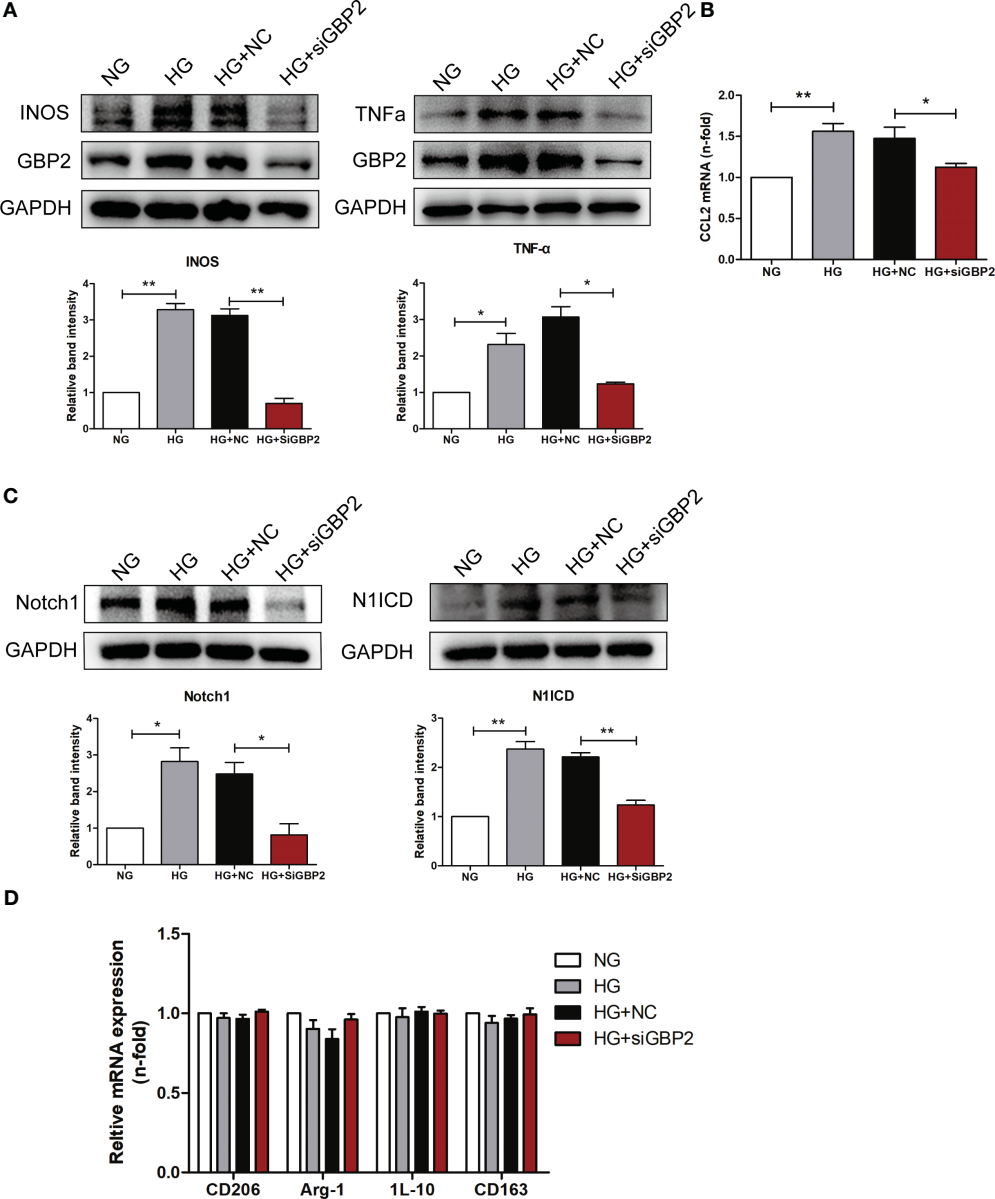
GBP2 induced M1 macrophage polarization. **(A)** Immunoblot analysis of M1 macrophage polarization marker INOS and pro-inflammatory cytokines TNF-α level in THP-1 cells transfected with siGBP2 under HG conditions, and GAPDH was used as an internal control. **(B)** Gene expression of M1-associated molecule CCL2 was determined by quantitative RT-PCR, and GAPDH was used as a loading control. **(C)** Notch1 and N1ICD protein levels in siGBP2-transfected THP-1 cells followed by HG treatment, and GAPDH was used as an internal control. **(D)** Gene expression of M2-associated molecules CD206, Arg-1, IL-10, and CD163 were determined by quantitative RT-PCR, and GAPDH was used as a loading control. NC: negative control siRNA. Data are representative of three independent experiments. Values are expressed as means ± SEM, *P < 0.05; **P <0.01.

Given the known correlation between the first three hub genes (CASP1, MS4A4A, and CD53) and macrophage-mediated inflammation, experiments were carried out to investigate the effect of GBP2. *In vivo*, F4/80 was increased in db/db mice, indicating the presence of macrophages in DN (**Figure 7A**). GBP2 was enhanced in the kidney tissues of DN patients and db/db mice, especially in the tubulointerstitium (**Figure 7B**). Further experiments verified that GBP2 co-localized with macrophage marker F4/80 and the co-location was increased in db/db mice (**Figure 7C**), demonstrating that GBP2 was also increased in macrophages not only in tubular cells. Consistently, the increased expression level of GBP2 was also validated in THP-1 cells exposed to high glucose (**Figure 8A**). Moreover, in high glucose-cultured THP-1 cells, the elevated GBP2 expression paralleled with M1 macrophage marker INOS (**Figure 8B**). Moreover, inhibiting GBP2 with its siRNA can reverse HG-mediated high expression of INOS, TNF-α, and CCL2 (**Figures 9A, B**). In addition, the down-regulation of GBP2 by siRNA was along with a decline in the Notch1 signal (**Figure 9C**). Activated notch1 signaling is one of the most common pathways that involve in M1 macrophage polarization. Our results indicated that GBP2 knockdown was accompanied by suppressed Notch1 signaling, demonstrating that the M1 polarization-promoting effect of GBP2 may be accomplished by activating the Notch1 signaling pathway. To explore whether GBP2 affects the expression of M2 macrophages, the mRNA expression levels of M2 macrophage markers (CD206, Arg-1, and CD163) and anti-inflammatory cytokines (IL-10) were detected by real-time PCR. The results showed that the expression of these M2 macrophage-associated molecules remained unchanged (**Figure 9D**), indicating that GBP2 promoted macrophages toward M1 macrophage polarization and produced no effect on M2 macrophage.

## Discussion

A total of 459 DEGs were obtained between renal tubulointerstitial tissue samples from DN patients and normal samples, which were dominantly involved in phagosome, complement and coagulation cascades, staphylococcus aureus infection, and pertussis. The inflammatory reaction plays a protective role in response to a harmful stimulus, while non-resolving inflammation can cause tissue injury (7), which has been demonstrated in DN progression (13, 14). Evidence is accumulating for the participation of immune dysfunction and complement activation in the tubulointerstitial lesions in DN (15–17). Activated macrophages have been proven to contribute to the occurrence and progression of DN (5, 17). GO functional analysis indicated that they were significantly enriched in collagen-containing extracellular matrix, response to interferon-gamma, zymogen activation, and protein processing. Accumulation of extracellular matrix and immunity and inflammation are the prominent characteristics of diabetic nephropathy (18, 19). Zymogen activation and protein processing partake in various cellular processes and maintain cellular homeostasis, the dysfunction of which is undoubtedly prone to the development of DN.

Immune infiltration analysis showed that multiple immune cells may involve in DN progression. Increasing immune cells, including monocytes, M1 macrophages, neutrophils, γδT cells, and plasma cells, may exert adverse effects during the disease. Nevertheless, decreasing immune cells, including regulatory T cells (Tregs), may play a renoprotective role in DN. As circulating pro-inflammatory cytokines and pro-inflammatory monocytes enhanced, monocytes were driven into the renal tissue and differentiated into macrophages. The degree of monocyte/macrophage infiltration is associated with disease severity (4). Neutrophils are known to produce large amounts of reactive oxygen species and proteases, resulting in local inflammation and tissue damage (20). Neutrophils serve as one of the first responders against inflammation or injury, whereas their role in the DN progression remains poorly defined. Some studies, however, support the involvement of neutrophils in the pathogenesis of DN. The spontaneous adhesion of neutrophils in type 2 diabetic patients was augmented, which correlated positively with albuminuria (21). The neutrophil-to-lymphocyte ratio has been proven a reliable predictive marker of early-stage DN and worse outcomes (22, 23). γδ T cells secreted IL-17A and may aggravate renal dysfunction and disease progression in experimental DN models by reducing podocyte number and increasing infiltrating inflammatory cells (24). In terms of plasma cells, CD19lo/+CD38+ plasma cells were found increased in the peripheral blood of DN patients and were closely associated with renal damage, including increased urinary albumin excretion and decreased glomerular filtration rate (25). The most important mechanism underlying the role of plasma cells in contributing to DN progression is autoantibody production, causing the formation and deposition of immune complexes which triggers the activation of complement and subsequent renal injury. Immune complexes containing oxLDL or AGE–LDL present in the circulation of Type 1 and Type 2 diabetic patients were intimately related to albuminuria (26, 27). Furthermore, the inhibition of AGE–LDL-containing immune complexes with aminoguanidine was accompanied by decreased proinflammatory cytokines (28). A decrease in regulatory T cells was affirmed by previous research in which reduced Tregs and Treg/Th17 ratio was observed in the patients with type 2 diabetic nephropathy compared to type 2 diabetic patients without nephropathy and normal controls (29). The emerging evidence has demonstrated the beneficial effect of Tregs on the development of DN (30, 31). More serious diabetic renal injury was observed in db/db mice with Tregs depletion, while adoptive transfer of Tregs alleviated these detrimental alternations (32). This renal protective role may be attributed to its anti-inflammatory effect against pro-inflammatory T helper 17 cells (29).

Mast cell accumulation and degranulation levels were found elevated as the disease progresses of DN, which participated in tubulointerstitial injury through the production of tryptase, chymase, TGF-β1, renin, and TNF-α (33). Disodium cromoglycate could inhibit the degranulation of mast cells and lighten tubulointerstitial collagen deposition in rats with type I diabetes (34). Chymase inhibitor TY-51469 suppressed oxidative stress in the renal podocytes of diabetic db/db mice, thereby decreasing albuminuria (35). This evidence revealed that mast cells were involved in DN progression. In the animal models of Type 1 and Type 2 diabetic nephropathy, an increase in the number of CD8+ T cells was mainly detected in the renal interstitium (36, 37). Furthermore, this phenomenon was also confirmed in individuals with type 2 diabetes (36). It was reported that the CD4+ cells were present in response to early renal damage and subsequently converted to CD8+ T cells at a late stage (38). CD8+ T cells contribute to the development and progression of DN. Mechanistically, on one hand, CD8+ T cells could recruit and activate neighboring macrophages by producing proinflammatory cytokines; on the other hand, CD8+ T cells caused diabetic kidney injury through direct cytotoxic effects (39). Previous studies have shown that mast cells and CD8+ T cells are increased in the kidney of DN. However, our study observed the opposite results, which may be due to different disease periods, relatively small sample sizes as well as sample heterogeneity.

Since macrophages play a crucial irreplaceable role in the occurrence and development, we focus on key genes closely related to macrophage-induced inflammation. The paleturquoise module most closely related to M1 macrophages was identified using WGCNA analysis. Among this paleturquoise module, a total of 17 genes holds the highest intra-module connectivity. To gain the differential genes with a strong association with the M1 phenotype, the intersection between these 17 genes and DEGs was performed. As a result, we selected 16 genes that were mainly involved in immunity and inflammation such as cellular defense response, activation of the innate immune response, positive regulation of innate immune response, and positive regulation of leukocyte adhesion to vascular endothelial cells. Enriched KEGG pathways included pertussis and leishmaniasis, which also revealed that non-resolving inflammation contributed to the development of DN. Subsequently, four hub genes, namely CASP1, MS4A4A, CD53, and GBP2 were identified using LASSO analysis. In DN patients, the expression levels of these four genes were significantly higher than in healthy individuals, which was validated by another dataset. Further, all these four genes achieved an AUC >0.8, indicating a high diagnostic value for DN. Clinically, high serum creatine, high proteinuria, and low GFR portend more severe disease and poorer prognosis. Based on the association between these hub genes and these three indicators, we found that all of these four genes were associated with illness severity and poor outcome, demonstrating that these genes may serve as potential biomarkers for monitoring the condition and evaluating the prognosis.

It has been proved that macrophages are not only involved in DN progression, but closely associated with DN prognosis. Exposed to different stimuli, macrophages can differentiate toward either M1 macrophages or M2 macrophages. M1 phenotype is induced by microbial molecules or LPS/IFN-γ and M2 phenotype is generated by IL-4 and IL-13. F4/80 or CD68 is applied as an indicator of pan-macrophage, INOS is for the M1 phenotype and CD206 for the M2 phenotype. Furthermore, these two types of phenotypes are present at different phases and play an opposite role. Accumulating evidence revealed that injury-site macrophages were predominantly M1 macrophages. Macrophage-specific cyclooxygenase-2 knockout DN mice exhibited elevated M1 phenotypes polarization and more serious kidney injury (10). It was reported that excessive activation of the notch1 pathway in macrophages promotes the polarization of macrophages towards a proinflammatory M1 phenotype. IL37 could mitigate the polarization of the M1 phenotype by suppressing the Notch1 signaling pathway (40). In diabetic nephropathy, ectopic activation of the Notch1 pathway drove macrophage differentiation into a pro-inflammatory M1 subtype. Additionally, the repression of the Notch1 signaling in macrophages mitigated the pathological alterations including inflammatory injury and fibrosis (6).

Among these four hub genes, CASP1, MS4A4A, and CD53 have been explored in macrophage-mediated inflammatory reactions. CASP1(Caspase1) is known as the downstream enzyme of NLRP3 inflammasome and subsequently is activated, which is closely associated with pyroptosis and inflammation (41). Active caspase1 could cleave the immature precursors of downstream inflammatory cytokines interleukin (IL)-1β and IL-18, triggering an inflammatory programmed cell death called pyroptosis (42). Carnosine could lighten podocyte injury in DN by suppressing caspase1-induced pyroptosis (43). Caspase1 aggravated hepatocellular injury in steatotic liver exposed to ischemia-reperfusion injury by mediating pyroptosis (44). In addition, caspase-1 was reported to aggravate tubulointerstitial fibrosis in DN via mediating pyroptosis and inflammation. In addition, caspase1 was associated with lipid and glucose metabolism (45). It has been found that caspase1 knockout mice exhibited obesity (46). MS4A4A, a tetraspan molecule regulated by M2-like stimuli, has been widely viewed as an M2 macrophage marker (47, 48). It has been demonstrated that MS4A4A was expressed in macrophages and enacted anti-tumor functions (48). In allergic inflammation, MS4A4A was involved in M2 macrophage polarization by promoting the expression of Arginase1 (49). CD53, a member of the tetraspanin family, is highly expressed by multiple immune cells, including macrophages, B cells, CD8 T cells, CD4 T cells, and natural killer cells (50). It has been reported that CD53 contributed to the transendothelial migration of neutrophils and subsequent inflammatory response (51). GBP2 belongs to the GTPase family and is significantly elevated after IFN-γ stimulation, which plays a vital role in host immunity against viral infection (52). GBP2 not only plays an indispensable role in the onset and progression of multiple tumors, but also exhibits a strong prognostic value. However, the function of GBP2 differs in different cancer types. For example, GBP2 accelerated the proliferation and migration of glioma via KIF22/EGFR pathway (53). Likewise, highly expressed GBP2 contributed to the invasion of glioblastoma multiforme through Stat3/FN1 signaling pathway (54). However, the expression of GBP2 was decreased in colorectal cancer, which gave rise to poor prognosis and metastasis (55). In addition, GBP2 was closely associated with the activation of caspase-11 inflammasomes, hence facilitating the occurrence of sepsis (56). Among these four hub genes screened, CASP1, MS4A4A, and CD53 have been explored in macrophage-mediated inflammatory reactions. Nevertheless, the association between GBP2 and macrophage is unknown and GBP2 has not been reported in diabetic nephropathy. Therefore, we focused on it and conducted experiments to validate the role of GBP2 in M1 polarization and the potential mechanism.

Similar to the effect of LPS, the expression of GBP2 was elevated in THP-1 cells under HG conditions, which went along with increased M1 marker INOS. Likewise, up-regulated GBP2 was observed in db/db mice and DN patients. To further elucidate the role of GBP2, small interfering RNA (siGBP2) was employed to silence GBP2. The results showed that the silence of GBP2 prevented macrophages from polarizing into pro-inflammatory M1 phenotypes. In addition, inhibiting GBP2 could reduce M1 macrophage-produced pro-inflammatory cytokines TNF-α and CCL2, but exerted no effect on M2 macrophage markers and anti-inflammatory cytokines. Furthermore, it has been known that activated the Notch1 signaling pathway plays a critical role in M1 macrophage polarization. Along with the inhibition of GBP2, the Notch1 pathway was suppressed, indicating GBP2 may facilitate M1 polarization through the activation of the canonical Notch pathway. Our previous studies have confirmed that Notch1 intracellular domain (N1ICD) is separated from the Notch1 extracellular domain (N1ECD) and subsequently translocates to the nucleus where it initiates target gene expression (12). GBP2 may impact notch1 activation directly or indirectly including promoting the nuclear translocation of N1ICD through their direct interaction or modulating N1ICD acetylation in an indirect manner (57, 58). In the following experiments, our work will revolve around the mode of interaction between GBP2 and Notch1 and their potential binding sites. Collectedly, GBP2 may be a candidate target for alleviating renal tubular injury. Further basic experiments and clinical trials are entailed to elucidate the role of GBP2.

Our study has several strengths. The findings in the present study are the first to elucidate the critical genes which are associated with pro-inflammatory M1 macrophages in DN via transcriptomic analysis. In addition, multiple bioinformatic methods, including CIBERSORT, WGCNA, and LASSO regression analysis, are employed. Of importance, the results from bioinformatics analysis are validated *in vitro* and *in vivo* experiments. Hence, our findings may be more accurate and credible. Notwithstanding these merits, the study has several limitations. More experiments are entailed to clarify the mechanisms by which GBP2 regulates the Notch1 signaling pathway. Moreover, Clinical trials are needed to confirm our results for better translation into the clinic.

## Conclusion

Integrating the DEGs, WGCNA, and LASSO regression, M1 macrophage-associated four hub genes (CASP1, MS4A4A, CD53, and GBP2) were determined. For clinical significance, all these genes showed prime diagnostic effectiveness and a good prognostic value. We focused on GBP2 and demonstrated it facilitates macrophage M1 polarization through the activation of the notch1 pathway, thereby providing a potential therapeutic target.

## Data availability statement

The original contributions presented in the study are included in the article/**Supplementary Materials**. Further inquiries can be directed to the corresponding author.

## Ethics statement

The studies involving human participants were reviewed and approved by Medical Ethics Committee of Second Xiangya Hospital, Central South University. The patients/participants provided their written informed consent to participate in this study. The animal study was reviewed and approved by Medical Ethics Committee of Second Xiangya Hospital, Central South University.

## Author contributions

XL and LX conceived and designed the study. XL performed the experiments and drafted the manuscript. LX performed the analysis and revised the manuscript. JL and MZ helped draft the manuscript and were involved in the study discussion. KY, SZ, and YL helped compiled and analyzed data. XY, CZ, and WW refined the manuscript. All authors contributed to the article and approved the submitted version.
